# Incidence and Predictors of Clinical Outcomes in Real‐Life Patients With Atrial Fibrillation Treated With Oral Factor Xa Inhibitors: The Follow‐Up Results of the ANATOLIA‐AF Study

**DOI:** 10.1002/clc.70088

**Published:** 2025-01-27

**Authors:** Umut Kocabaş, Isil Ergin, Sadi Can Sönmez, Veysel Yavuz, Selda Murat, Ibrahim Halil Özdemir, Ömer Genç, Haşim Tüner, Bengisu Keskin Meriç, Onur Aslan, Ahmet Dal, Uğur Taşkın, Taner Şen, Ersin İbişoğlu, Aslan Erdoğan, Mehmet Özgeyik, Mevlüt Demir, Örsan Deniz Urgun, Mustafa Doğduş, Sinem Çakal, Sercan Çayırlı, Arda Güler, Dilay Karabulut, Onur Dalgıç, Bektaş Murat, Umut Karabulut, Gülsüm Meral Yılmaz Öztekin, Halil İbrahim Biter, Ümit Yaşar Sinan, Veysel Özgür Barış, Mehmet Kaplan, Cihan Altın, Tarık Kıvrak

**Affiliations:** ^1^ Department of Cardiology Başkent University Izmir Hospital Izmir Türkiye; ^2^ Department of Public Health Faculty of Medicine, Ege University Izmir Türkiye; ^3^ Department of Cardiology Akhisar State Hospital Manisa Türkiye; ^4^ Department of Cardiology, Faculty of Medicine Eskişehir Osmangazi University Eskişehir Türkiye; ^5^ Department of Cardiology Manisa City Hospital Manisa Türkiye; ^6^ Department of Cardiology Ağrı Training and Research Hospital Ağrı Türkiye; ^7^ Department of Cardiology Hakkari State Hospital Hakkari Türkiye; ^8^ Department of Cardiology Babaeski State Hospital Kırklareli Türkiye; ^9^ Department of Cardiology Mersin City Hospital Mersin Türkiye; ^10^ Department of Cardiology, Faculty of Medicine Izmir University of Economics Izmir Türkiye; ^11^ Department of Cardiology, Faculty of Medicine Kütahya Health Sciences University Kütahya Türkiye; ^12^ Department of Cardiology Başakşehir Çam and Sakura City Hospital Istanbul Türkiye; ^13^ Department of Cardiology Eskişehir City Hospital Eskişehir Türkiye; ^14^ Department of Cardiology Kozan State Hospital Adana Türkiye; ^15^ Department of Cardiology Uşak University Training and Research Hospital Uşak Türkiye; ^16^ Department of Cardiology, Haseki Training and Research Hospital Health Sciences University Istanbul Türkiye; ^17^ Department of Cardiology, Faculty of Medicine Adnan Menderes University Aydın Türkiye; ^18^ Department of Cardiology Istanbul Mehmet Akif Ersoy Thoracic and Cardiovascular Surgery Training and Research Hospital Istanbul Türkiye; ^19^ Department of Cardiology Istanbul Bakirköy Dr Sadi Konuk Training and Research Hospital Istanbul Türkiye; ^20^ Department of Cardiology Kardiya Medical Center Izmir Türkiye; ^21^ Department of Cardiology Istanbul Acıbadem International Hospital Istanbul Türkiye; ^22^ Department of Cardiology, Antalya Training and Research Hospital Health Sciences University Antalya Türkiye; ^23^ Department of Cardiology Istanbul University‐Cerrahpasa Institute of Cardiology Istanbul Türkiye; ^24^ Department of Cardiology Dr Ersin Arslan Training and Research Hospital Gaziantep Türkiye; ^25^ Department of Cardiology Gaziantep University Medical School Gaziantep Türkiye; ^26^ Department of Cardiology Firat University Hospital Elazig Türkiye

**Keywords:** atrial fibrillation, bleeding, factor Xa inhibitors, mortality, outcome, stroke

## Abstract

**Objective:**

The main objective of this study is to determine the incidence and predictors of clinical outcomes in patients with AF treated with factor Xa inhibitors in a real‐world setting.

**Methods:**

The present study was a multicentre and observational study that included patients with AF who were treated with factor Xa inhibitors. The primary outcome was the composite of ischemic stroke, TIA, systemic embolism, major bleeding, and all‐cause mortality.

**Results:**

A total of 1162 patients from 26 cardiology centers were included in this study, with a median age of 72 years. During the median 12‐month follow‐up period, the primary outcome occurred in 195 patients (16.8%). Treatment with rivaroxaban compared with apixaban and edoxaban showed a lower rate of ischemic stroke, TIA, and/or systemic embolism (2.2% vs. 4.7% vs. 6.5%, respectively, *p* = 0.014). The major bleeding rate was similar between all three factor Xa inhibitors. The all‐cause mortality rate in the rivaroxaban group was lower compared with the apixaban and edoxaban groups (9.8% vs. 15.1% vs. 12.4%, respectively, *p* = 0.042). Overall, the frequency of primary outcome was 13.8%, 19.6%, and 20.6% for patients treated with rivaroxaban, apixaban, and edoxaban, respectively (*p* = 0.019). Older age, male sex, low body weight, high bleeding risk, heart failure, hypertension, liver failure, and treatment with apixaban 2.5 mg b.i.d. were independently associated with the development of primary outcome.

**Conclusion:**

The follow‐up data from the ANATOLIA‐AF study provides detailed data about the incidence and independent predictors of adverse clinical outcomes in patients with AF treated with factor Xa inhibitor treatment.

## Summary

- The net clinical outcome and all‐cause mortality rates were 16.8% and 12.0%, respectively.
- The net clinical outcome and all‐cause mortality rates were significantly lower in patients treated with rivaroxaban than those treated with apixaban or edoxaban.
- Older age, male sex, low body weight, high bleeding risk, hypertension, heart failure, liver failure, and apixaban 2.5 mg B.I.D. treatment were independently associated with the occurrence of net clinical outcome.

## Graphical abstract

The net clinical outcome results and independent predictors of net clinical outcomes.

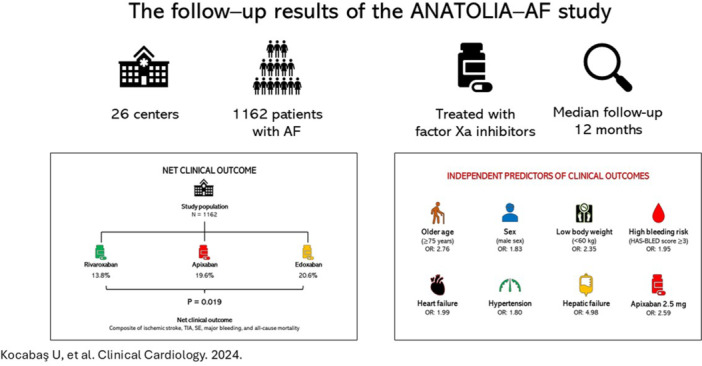

## 1 Introduction

Atrial fibrillation (AF) is the most prevalent sustained cardiac arrhythmia in clinical practice, with a global prevalence ranging from 2% to 4% [1]. Patients with AF have a fivefold higher risk of stroke or systemic embolism and cardiovascular death compared to an age‐ and sex‐matched general population [1, 2]. Oral anticoagulant therapy, comprising vitamin K antagonists (VKAs) and direct oral anticoagulants (DOACs), is the cornerstone of AF management to prevent stroke and systemic embolism [3].

Direct oral anticoagulants, particularly factor Xa inhibitors, were developed to overcome limitations of VKAs, including delayed onset and offset of anticoagulation, need for close monitoring, narrow therapeutic window, and drug‐food interactions [3]. Factor Xa inhibitors, rivaroxaban, apixaban, and edoxaban, have demonstrated non‐inferiority or superiority to warfarin in the prevention of stroke and systemic embolism in randomized clinical trials (RCTs) [4, 5, 6]. Based on this evidence, current guidelines recommend DOACs as first‐line therapy for the prevention of stroke and systemic embolism in patients with AF without a mechanical heart valve or moderate‐to‐severe mitral stenosis [1, 7].

In evidence‐based medicine, RCTs are pivotal in the assessment of the safety and efficacy of novel drugs. However, these trials have strict inclusion and exclusion criteria limiting the generalizability of the results to the general population. Real‐world studies more accurately reflect routine clinical practice, and these studies are essential to fill the gaps in RCTs with highly selected participants [8]. Although factor Xa inhibitors have proved their safety and efficacy against VKAs in RCTs, patients with AF and other comorbidities comprising older age, frailty, low body weight or obesity, chronic kidney disease, severe anemia, and chronic liver failure are often underrepresented in the RCTs. In addition, real‐world data on clinical outcomes and associated factors in patients with AF treated with factor Xa inhibitors are conflicting [9, 10, 11, 12]. Therefore, it is essential to determine the incidence and predictors of clinical outcomes in patients with AF treated with oral factor Xa inhibitors. The main objective of this study is to determine the incidence and predictors of clinical outcomes in patients with AF treated with factor Xa inhibitors in a real‐world setting.

## 2 Methods

### 2.1 Study Design and Population

The study population was derived from the ANATOLIA‐AF study and the detailed study protocol has been previously published [13]. Briefly, the ANATOLIA‐AF study is a multicenter and observational study that included unselected outpatients with AF. The baseline study population included 2782 consecutive outpatients (aged ≥18 years old) enrolled at 41 cardiology centers from January 2021 to May 2021. We excluded 182 patients with a mechanical heart valve or moderate‐to‐severe mitral stenosis, 535 patients who were not taking a DOAC and 120 patients who were receiving dabigatran at baseline, and 783 patients with missing baseline clinical and/or follow‐up data. Of the enrolled patients from 26 centers, 1162 were eligible for inclusion and the final analysis of this study. Strobe diagram of the study summarized in Figure S1.

### 2.2 Data Collection and Definitions

Patients with AF were diagnosed based on documentation on 12‐lead electrocardiography and/or 24‐h Holter electrocardiography recording. Baseline socio‐demographic data at the time of the first visit were self‐reported by study participants. Baseline patient clinical and laboratory data including previous medical history and comorbidities, history of stroke and bleeding, anticoagulant treatments and concurrent antiplatelet medications, kidney function (serum creatinine levels and estimated glomerular filtration rate [GFR]) were collected at the first visit and recorded in a case report form by study investigators with standardized definitions for all fields.

Estimated GFR was calculated for each patient from the serum creatinine level using the Cockcroft–Gault equation [14], a method that was used in the pivotal trials of DOACs [4, 5, 6]. CHA_2_DS_2_‐VASc (chronic heart failure or left ventricular dysfunction, hypertension, age ≥ 75 or 65–74 years, diabetes, history of stroke and/or systemic embolism, vascular disease, and sex) and HAS‐BLED (hypertension, renal failure and/or liver failure, history of stroke, bleeding history, labile international normalized ratio, age >65 years, concomitant drug use that predisposed to bleeding, and/or excessive alcohol use) scores were calculated for each study patient to assess thrombotic and bleeding risk [1]. High ischemic stroke and/or systemic embolism risk defined as CHA_2_DS_2_‐VASc score ≥3 in females or ≥ 2 in males, and high bleeding risk defined as HAS‐BLED ≥ 3 [1].

Major bleeding was defined as fatal bleeding, symptomatic bleeding in a critical organ or site (intracranial, intraocular, retroperitoneal, pericardial, and intraarticular), compartment syndrome intramuscular bleeding, overt bleeding leading to a decrease in hemoglobin level to at least 2.0 g/L or the need for transfusion of at least two units of erythrocyte suspension [15]. Clinically relevant nonmajor bleeding (CRNM) was defined as a clinically overt bleeding that did not meet the criteria for major bleeding but was associated with medical intervention, unscheduled contact with a physician, or interruption of anticoagulant treatment [16].

### 2.3 DOACs Dosage

Patients prescribed rivaroxaban 20 mg o.d., apixaban 5 mg b.i.d., or edoxaban 60 mg o.d. were considered to be receiving a standard dose; rivaroxaban 15 mg o.d., apixaban 2.5 mg b.i.d., and edoxaban 30 mg o.d. were considered as reduced doses. The criteria proposed by the summaries of product characteristics (SmPCs) of DOACs and current AF guidelines were used to determine the appropriate dose [1]. The study participants were classified as follows: (1) appropriate dose of DOAC (receiving an appropriate standard dose or appropriate reduced dose of DOAC); (2) inappropriate low dose of DOAC (receiving a reduced dose of DOAC without dose reduction criteria); and (3) inappropriate standard dose of DOAC (receiving a standard dose of DOAC despite the presence of dose reduction criteria) [13].

### 2.4 Follow‐Up

After enrolment, study participants were followed up in cardiology outpatient clinics. Treating physicians followed up patients at least every 6 months after enrollment. Additionally, some participants were followed up more frequently by their physician according to the physician's discretion. Follow‐up data retrospectively obtained from case report forms filled by study investigators or medical records of the hospital. For those patients without follow‐up in cardiology outpatient clinics, follow‐up data including ischemic stroke and/or transient ischemic attack (TIA) and/or systemic embolism, major and/or CRNM and/or minor bleeding and its localization, and death from any cause were obtained from telephone interviews with patients or patients' families.

### 2.5 Study Outcomes

The primary outcome was the risk‐benefit balance between major bleeding, thromboembolism, and mortality as reflected by the “net clinical outcome.” The net clinical outcome is defined as the composite outcome of ischemic stroke, TIA, systemic embolism, major bleeding, and/or all‐cause mortality. We also separately analyzed the safety outcomes including major bleeding and the composite outcome of major and/or CRNM bleeding, and the efficacy outcomes including the composite outcome of ischemic stroke and/or TIA and/or systemic embolism; and unadjusted all‐cause mortality rate for each factor Xa inhibitor. Study outcomes are evaluated and reported by study investigators.

### 2.6 Statistical Analysis

Data for continuous variables were summarized with a mean (±standard deviation) or median (interquartile range [IQR]) depending on the normality of distribution tested with a one‐sample Kolmogorov–Smirnov test. Percentages were used to summarize the data for categorical variables. For the comparison of factor Xa inhibitors, standard and reduced doses of factor Xa inhibitors or outcomes (clinical, safety, efficacy); the Student *t*‐test, MWU, ANOVA, and Kruskal–Wallis tests were used when appropriate for evaluating the group differences for continuous variables. The Pearson *χ*2 or Fisher's exact tests were used for categorical comparisons. Variables detected to show significant relation in bivariate analysis were checked for collinearity. Any detection of a strong correlation (Pearson correlation coefficient >0.60 with a *p*‐value < 0.05) leads to the exclusion of one of the two variables, from the Model (i.e., “major and/or CRNM bleeding” had a correlation coefficient of 0,625 with “major bleeding” and 0,712 with “history of GI bleeding.” “Major and/or CRNM bleeding” was selected for the model). Variables detected to be in relation with clinical, safety or efficacy outcomes in bivariate analysis were included in the logistic regression analysis for each outcome. Unadjusted odds ratios (ORs) and their 95% confidence intervals were presented for each variable in the univariate analysis column, while adjusted ORs were presented under multivariate analysis with the inclusion of all significantly related variables of the model. All statistical analyses were performed using SPSS version 23 for Windows (SPSS Inc., Chicago, IL, USA). *p‐*value < 0.05 was considered significant.

The study was conducted in accordance with the principles of the Declaration of Helsinki, and all patients gave written informed consent to participate. This study was approved by the local ethics committee (Project No. KA20/463 and E‐94603339‐604.01.02‐1547).

## 3 Results

### 3.1 Study Population

A total of 1162 patients with AF treated with factor Xa inhibitors were included in the present study, with a median age of 72 years (range: 28–96 years) and 59.6% female patients. The mean CHA_2_DS_2_‐VASc and HAS‐BLED scores of the study population were 3.8 ± 1.5 and 1.5 ± 1.0, respectively. The most common comorbidities were hypertension, chronic heart failure, anemia, and coronary artery disease. Overall, 16.1% of patients had a history of ischemic stroke and/or TIA, and 7.6% of them had a history of major and/or CRNM bleeding. The mean GFR was 71 ± 24 mL/min/1.73 m^2^, and 32.6% of patients had a GFR < 60 mL/min/1.73 m^2^. Among the study population, 122 patients (10.5%) were receiving concomitant antiplatelet therapy including aspirin, clopidogrel, ticagrelor, or prasugrel (Supporting Information S1: Table 1).

### 3.2 Factor Xa Inhibitors

The most prescribed factor Xa inhibitor was rivaroxaban (50.7%), followed by apixaban (34.7%), and edoxaban (14.6%). The standard dose of factor Xa inhibitors was prescribed to 869 patients (74.9%) and the reduced dose to 292 patients (25.1%). According to the SmPC of factor Xa inhibitors, 79.2% of the study population received the appropriate dose of factor Xa inhibitors and 20.8% of them received an inappropriate dose. Among patients receiving an inappropriate dose of factor Xa inhibitors, 15.2% received an inappropriately low dose, and 5.6% received an inappropriately standard dose (Supporting Information S1: Table 1).

### 3.3 Baseline Demographic and Clinical Characteristics of the Study Population According the Factor Xa Inhibitors

Among the study population, patients who were receiving apixaban were significantly older than those receiving rivaroxaban or edoxaban. The risk of ischemic stroke and systemic embolism as reflected by the CHA_2_DS_2_‐VASc score was higher in patients who were administered apixaban than those administered rivaroxaban or edoxaban. The proportion of patients with a history of ischemic stroke or systemic embolism and a history of intracranial hemorrhage was higher in the edoxaban group, whereas chronic kidney disease was more prevalent in patients receiving apixaban. While the prescription of inappropriately low doses of factor Xa inhibitors was more common in the rivaroxaban and apixaban groups, the prescription of inappropriately standard doses of factor Xa inhibitors was significantly higher in patients with edoxaban treatment (Supporting Information S1: Table 2).

### 3.4 Net Clinical Outcomes

During the median 12 months (IQR: 2) follow‐up period, 43 patients (3.7%) had ischemic stroke, TIA, and/or systemic embolism and 36 patients (3.1%) had major bleeding. A total of 140 (12.0%) patients died. Overall, the frequency of net clinical outcomes (composite of ischemic stroke, TIA, systemic embolism, major bleeding, and/or all‐cause mortality) was 16.8% (195 patients) among the study population (Table 1).

**TABLE 1:** The net clinical outcome, the safety outcomes, and the efficacy outcomes according to the factor Xa inhibitors.

	Overall (*N* = 1162)	Rivaroxaban (*N* = 589)	Apixaban (*N* = 403)	Edoxaban (*N* = 170)	*p‐*value
Net clinical outcome^[Link]^, ^a^, *n* (%)	195 (16.8)	81 (13.8)	79 (19.6)	35 (20.6)	0.019
Safety outcomes, *n* (%)					
Major bleeding	36 (3.1)	20 (3.4)	13 (3.2)	3 (1.8)	0.54
CRNM bleeding	56 (4.8)	24 (4.1)	22 (5.5)	10 (5.9)	0.47
Major and/or CRNM bleeding	94 (8.1)	46 (7.8)	33 (8.2)	15 (8.8)	0.90
Minor bleeding	287 (24.7)	140 (23.8)	108 (26.8)	39 (22.9)	0.47
Intracranial hemorrhage	8 (0.7)	2 (0.35)	2 (0.51)	4 (2.4)	0.017
Gastrointestinal bleeding	69 (5.9)	33 (5.7)	27 (6.8)	9 (5.4)	0.70
Efficacy outcomes, *n* (%)					
Ischemic stroke and/or TIA	42 (3.6)	13 (2.2)	18 (4.5)	11 (6.5)	0.017
Ischemic stroke and/or TIA and/or systemic embolism	43 (3.7)	13 (2.2)	19 (4.7)	11 (6.5)	0.014
Myocardial infarction	29 (2.5)	14 (2.4)	12 (3.0)	3 (1.8)	0.67
All‐cause mortality, *n* (%)	140 (12.0)	58 (9.8)	61 (15.1)	21 (12.4)	0.042

*Note:* Safety outcomes: Composite of major bleeding and/or CRNM bleeding; Efficacy outcomes: Composite of ischemic stroke, TIA, and/or systemic embolism.

Abbreviations: CRNM = clinically relevant non‐major; SE = systemic embolism; TIA = transient ischemic attack.

^a^ Net clinical outcome: Composite of ischemic stroke, TIA, systemic embolism, major bleeding, and/or all‐cause mortality.

**FIGURE 1:**
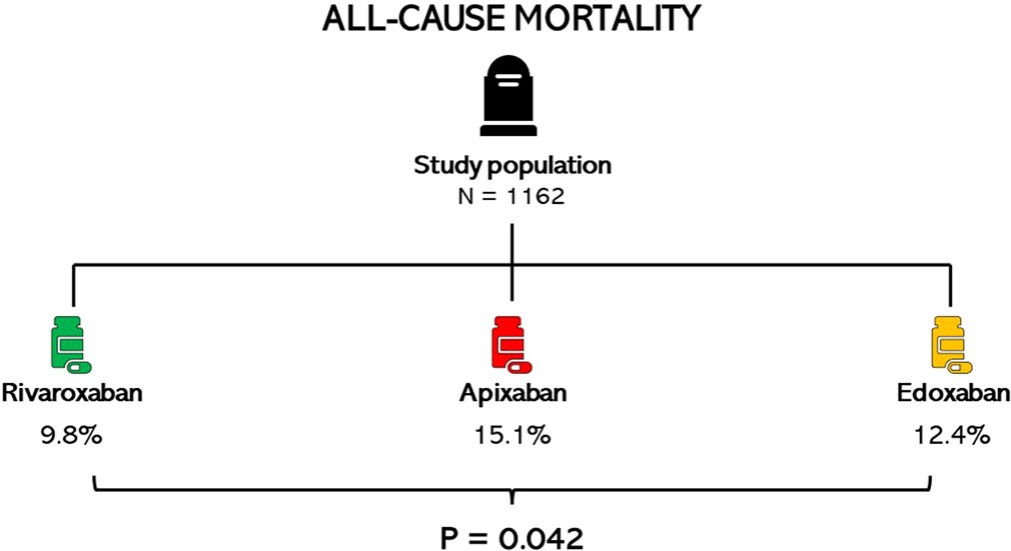
The all‐cause mortality rates of factor Xa inhibitors.

Treatment with rivaroxaban compared with apixaban and edoxaban showed a lower rate of ischemic stroke, TIA, and/or systemic embolism during the follow‐up period (2.2% vs. 4.7% vs. 6.5%, respectively, *p* = 0.014). The major bleeding rate was similar between all three factor Xa inhibitors. The all‐cause mortality rate in the rivaroxaban group was lower compared with the apixaban and edoxaban group (9.8% vs. 15.1% vs. 12.4%, respectively, *p* = 0.042) (Figure 1). Overall, the frequency of net clinical outcomes was 13.8% for patients treated with rivaroxaban, 19.6% for patients treated with apixaban, and 20.6% for patients treated with edoxaban (*p* = 0.019) (Table 1, graphical abstract and Figure S2).

**FIGURE 2:**
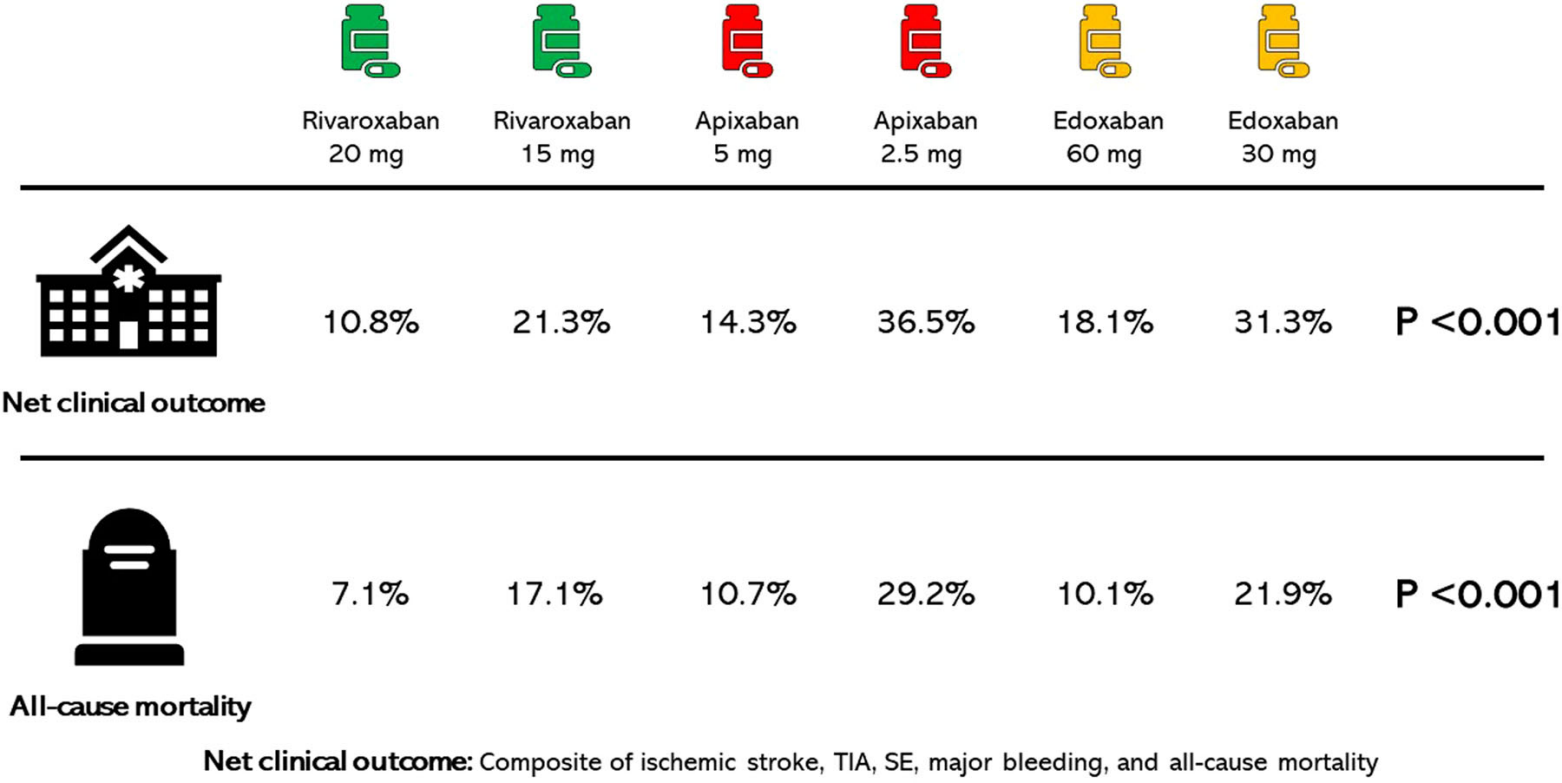
The net clinical outcome and all‐cause mortality rates of standard and reduced doses of factor Xa inhibitors.

There was a significant difference between standard and reduced doses of each factor Xa inhibitor regarding net clinical outcomes. While the rate of net clinical outcomes was 21.3% for patients receiving rivaroxaban 15 mg o.d., the same rate was 36.5% and 31.3% for patients receiving apixaban 2.5 mg b.i.d. and edoxaban 30 mg o.d., respectively (*p* < 0.001). Similarly, the all‐cause mortality rate was 17.1% for patients treated with rivaroxaban 15 mg o.d., 29.2% for patients treated with apixaban 2.5 mg b.i.d., and 21.9% for patients treated with edoxaban 30 mg o.d., respectively (*p* < 0.001) (Table 2 and Figure 2).

**TABLE 2:** The net clinical outcome, the safety outcomes, and the efficacy outcomes according to the standard and reduced doses of factor Xa inhibitors.

	Rivaroxaban 20 mg OD	Rivaroxaban 15 mg OD	Apixaban 5 mg BID	Apixaban 2.5 mg BID	Edoxaban 60 mg OD	Edoxaban 30 mg OD	*p‐*value
Net clinical outcome^a^, *n* (%)	46 (10.8)	35 (21.3)	44 (14.3)	35 (36.5)	25 (18.1)	10 (31.3)	<0.001
Safety outcomes, *n* (%)							
Major bleeding	12 (2.8)	8 (4.9)	7 (2.3)	6 (6.2)	2 (1.5)	1 (3.1)	0.22
CRNM bleeding	10 (2.4)	14 (8.5)	13 (4.2)	9 (9.4)	8 (5.8)	2 (6.2)	0.008
Major and/or CRNM bleeding	30 (7.1)	16 (9.8)	24 (7.8)	9 (9.4)	12 (8.7)	3 (9.4)	0.90
Minor bleeding	87 (20.5)	53 (32.3)	307 (24.1)	34 (35.4)	31 (22.5)	8 (25)	0.008
Intracranial hemorrhage	1 (0.24)	1 (0.62)	2 (0.67)	0 (0.0)	4 (2.94)	0 (0.0)	0.036
Gastrointestinal bleeding	18 (4.3)	15 (9.3)	16 (5.3)	11 (11.7)	6 (4.1)	3 (9.7)	0.033
Efficacy outcomes, *n* (%)							
Ischemic stroke and/or TIA	8 (1.9)	5 (3.0)	10 (3.3)	8 (8.3)	8 (5.8)	3 (9.4)	0.010
Ischemic stroke and/or TIA and/or systemic embolism	8 (1.9)	5 (3.0)	10 (3.3)	9 (9.4)	8 (5.8)	3 (9.4)	0.004
Myocardial infarction	10 (2.4)	4 (2.4)	8 (2.6)	4 (4.2)	1 (0.7)	2 (6.3)	0.44
All‐cause mortality, *n* (%)	30 (7.1)	28 (17.1)	33 (10.7)	28 (29.2)	14 (10.1)	7 (21.9)	<0.001

*Note:* Safety outcomes: Composite of major bleeding and/or CRNM bleeding; Efficacy outcomes: Composite of ischemic stroke, TIA, and/or systemic embolism.

Abbreviations: CRNM = clinically relevant non‐major, SE = systemic embolism, TIA = transient ischemic attack.

^a^ Net clinical outcome: Composite of ischemic stroke, TIA, systemic embolism, major bleeding, and/or all‐cause mortality.

Compared with patients without clinical outcomes, those with clinical outcomes were significantly older, more likely to be male, more likely to have a history of stroke and/or TIA, chronic heart failure, hypertension, chronic kidney disease, anemia, chronic liver failure, more likely to have a history of major and/or CRNM bleeding and gastrointestinal bleeding, higher CHA_2_DS_2_‐VASc and HAS‐BLED scores, lower body weight and body mass index, GFR, and hemoglobin levels. The prescription of inappropriately low or standard doses of factor Xa inhibitors was significantly higher in patients with clinical outcomes (Supporting Information S1: Table 3).

### 3.5 Safety Outcomes

Among the population, the rate of major and/or CRNM bleeding (=safety outcomes) was 8.1% (94 patients) during the follow‐up period (Table 1). The major and/or CRNM bleeding rate was similar between all three factor Xa inhibitors and there were no significant differences in their standard and reduced doses (Tables 1 and 2). Patients with major and/or CRNM bleeding were significantly older, more likely to have hypertension, anemia, and a history of bleeding, higher CHA_2_DS_2_‐VASc and HAS‐BLED scores, and lower hemoglobin levels compared with patients without major and/or CRNM bleeding (Supporting Information S1: Table 4).

### 3.6 Efficacy Outcomes

Overall, the frequency of ischemic stroke, TIA, and/or systemic embolism (=efficacy outcomes) was 3.7% (43 patients) during the follow‐up period (Table 1). The ischemic stroke, TIA, and/or systemic embolism rate in the rivaroxaban group was lower compared with the apixaban and edoxaban group (2.2% vs. 4.7% vs. 6.5%, respectively, *p* = 0.014). While the rate of ischemic stroke, TIA, and/or systemic embolism was 3.0% for patients receiving rivaroxaban 15 mg o.d., the same rate was 9.4% and 9.4% for patients receiving apixaban 2.5 mg b.i.d. and edoxaban 30 mg o.d., respectively (*p* = 0.004) (Table 2). Patients with ischemic stroke, TIA, and/or systemic embolism were more likely to have a history of stroke and/or TIA, higher CHA_2_DS_2_‐VASc, and HAS‐BLED scores (Supporting Information S1: Table 5).

### 3.7 Clinical Predictors of Net Clinical Outcomes

In the logistic regression model, the presence of advanced age (odds ratio [OR]: 2.01; 95% confidence interval [CI], 1.07–3.79, *p*‐value = 0.030 for patients between 65 and 74 years, and OR: 2.76; 95% CI, 1.43–5.33, *p*‐value = 0.002 for patients ≥75 years), male sex (OR: 1.83; 95% CI, 1.27–2.64, *p*‐value = 0.001), low body weight (≤60 kg) (OR: 2.35; 95% CI, 1.31–4.21, *p*‐value = 0.004), high bleeding risk (HAS–BLED score ≥ 3) (OR: 1.95; 95% CI, 1.24–3.08, *p*‐value = 0.004), chronic heart failure (OR: 1.99; 95% CI, 1.39–2.84, *p*‐value < 0.001), hypertension (OR: 1.80; 95% CI, 1.10–2.95, *p*‐value = 0.018), chronic liver failure (OR: 4.98; 95% CI, 1.24–19.88, *p*‐value = 0.023), and treatment with apixaban 2.5 mg b.i.d. (OR: 2.59; 95% CI, 1.13–5.92, *p*‐value = 0.023) were independently associated with the development of net clinical outcomes comprising ischemic stroke, TIA, systemic embolism, major bleeding, and/or all‐cause mortality among study population (Table 3, graphical abstract, and Figure S3).

**TABLE 3:** Univariate and multivariate analysis of the net clinical outcomes among study population^[Link]^, ^a^.

	Univariable analysis			Multivariable analysis		
Variable	Odds ratio	95% CI	*p* ‐value	Odds ratio	95% CI	*p* ‐value
Age group						
<65 years (reference)						
65–74 years	1.80	1.06–3.07	0.028	2.01	1.07–3.79	0.030
≥75 years	3.55	2.14–5.88	<0.001	2.76	1.43–5.33	0.002
Male sex	1.39	1.02–1.90	0.034	1.83	1.27–2.64	0.001
Body mass index (per 1 unit increase)	0.97	0.94–1.004	0.087	1.03	0.99–1.07	0.088
Low body weight^b^	2.40	1.57–3.68	<0.001	2.35	1.31–4.21	0.004
High stroke risk^c^	2.55	1.35–4.82	0.004	0.60	0.27–1.34	0.21
High bleeding risk^d^	3.18	2.18–4.65	<0.001	1.95	1.24–3.08	0.004
Previous stroke and/or TIA	1.74	1.19–2.54	0.004	1.47	0.94–2.29	0.089
Chronic heart failure	2.19	1.60–2.99	<0.001	1.99	1.39–2.84	<0.001
Hypertension	1.67	1.09–2.57	0.018	1.80	1.10–2.95	0.018
Chronic liver failure	5.06	1.45–17.66	0.011	4.98	1.24–19.88	0.023
Anemia	2.08	1.52–2.84	<0.001	1.31	0.91–1.88	0.13
History of minor bleeding	1.40	1.001–1.97	0.049	1.07	0.73–1.56	0.70
History of major and/or CRNM bleeding	2.38	1.47–3.86	<0.001	1.39	0.81–2.39	0.23
DOAC dosages						
Rivaroxaban 20 mg OD (reference)						
Rivaroxaban 15 mg OD	2.23	1.37–3.62	0.001	1.30	0.63–2.66	0.47
Apixaban 5 BID	1.37	0.88–2.14	0.15	1.13	0.70–1.84	0.59
Apixaban 2.5 BID	4.72	2.82–7.92	<0.001	2.59	1.13–5.92	0.023
Edoxaban 60 OD	1.82	1.07–3.09	0.027	1.71	0.97–3.01	0.059
Edoxaban 30 OD	3.74	1.67–8.39	0.001	1.92	0.72–5.09	0.18
Appropriate or inappropriate dosages						
Appropriate dose (reference)						
Inappropriate reduced dose	2.23	1.48–3.34	<0.001	0.94	0.48–1.82	0.86
Inappropriate standard dose	2.32	1.27–4.24	0.006	1.15	0.55–2.41	0.70
GFR group						
GFR ≥ 60 mL/min/1.73 m^2^ (reference)						
GFR 30—59 mL/min/1.73 m^2^	1.99	1.43–2.76	<0.001	1.06	0.69–1.62	0.77
GFR 15—29 mL/min/1.73 m^2^	3.33	1.61–6.88	0.001	1.02	0.40–2.57	0.96

*Note:* CHA2DS2‐VASc score system = congestive heart failure, hypertension, age ≥ 75 (2 points), diabetes, stroke (2 points), vascular disease, age 65–74, sex category (female). HAS‐BLED score system = uncontrolled hypertension, abnormal renal and liver function (1 point each), stroke, bleeding, labile international normalized ratios, elderly (age > 65 years), drugs or alcohol (1 point each) (concomitant use of antiplatelet agents or non‐steroidal anti‐inflammatory drugs, alcohol abuse).

Abbreviations: CI = confidence interval, CRNM = clinically relevant non‐major bleeding, DOAC = direct oral anticoagulant, GFR = glomerular filtration rate, TIA = transient ischemic attack.

^a^ Net clinical outcome: Composite of ischemic stroke, TIA, systemic embolism, major bleeding, and/or all‐cause mortality.

^b^ Low body weight = body weight ≤ 60 kg.

^c^ High stroke risk = CHA2DS2‐VASc score ≥ 3 (female) and CHA2DS2‐VASc score ≥ 2 (male).

^d^ High bleeding risk = HAS‐BLED score ≥ 3.

### 3.8 Clinical Predictors of Safety Outcomes

The presence of high bleeding risk (HAS‐BLED score ≥ 3) (OR: 1.81; 95% CI, 1.02–3.21, *p*‐value = 0.043), anemia (OR: 1.78; 95% CI, 1.12–2.83, *p*‐value = 0.014), history of minor bleeding (OR: 1.94; 95% CI, 1.23–3.06, *p*‐value = 0.004), and history of major and/or CRNM bleeding (OR: 2.77; 95% CI, 1.53–5.02, *p*‐value = 0.001) were associated with the development of major and/or CRNM bleeding among study population (Supporting Information S1: Table 6).

### 3.9 Clinical Predictors of Efficacy Outcomes

The history of stroke and/or TIA (OR: 3.09; 95% CI, 1.41–6.76, *p*‐value = 0.005) was associated with the development of ischemic stroke, TIA, and/or systemic embolism. Compared with rivaroxaban 20 mg o.d. treatment (reference group), treatment with apixaban 2.5 mg b.i.d. (OR: 4.64; 95% CI, 1.63–13.20, *p*‐value = 0.004), treatment with edoxaban 60 mg o.d. (OR: 3.03; 95% CI, 1.10–8.30, *p*‐value = 0.031), and treatment with edoxaban 30 mg o.d. (OR: 4.18; 95% CI, 1.01–17.27, *p*‐value = 0.048) were independent predictors of the development of ischemic stroke, TIA, and/or systemic embolism (Supporting Information S1: Table 7).

## 4 Discussion

The long‐term follow‐up data from the ANATOLIA‐AF study provides real‐life evidence for the incidence and predictors of net clinical outcomes in patients with AF treated with factor Xa inhibitors. The principal findings of this study are as follows: (i) the net clinical outcome and all‐cause mortality rates among the entire study population were 16.8% and 12.0%, respectively; (ii) the net clinical outcome and unadjusted all‐cause mortality rates were significantly lower in patients treated with rivaroxaban than those treated with apixaban or edoxaban; (iii) older age, male sex, low body weight, high bleeding risk, the presence of hypertension, chronic heart failure, and chronic liver failure, and apixaban 2.5 mg B.I.D. treatment were independently associated with the occurrence of net clinical outcomes.

The observed net clinical outcome rate in our study appears to corroborate the clinical impact of factor Xa inhibitors in real‐world practice, comparable to those observed in RCTs. The net clinical outcome rate reported 11.1% in the Apixaban versus Warfarin in Patients with Atrial Fibrillation trial during the median 1.8 years follow‐up period [5]. The Edoxaban versus Warfarin in Patients with Atrial Fibrillation trial demonstrated that the net clinical outcome rate was 18.9% in the edoxaban 60 mg arm and 17.8% in the edoxaban 30 mg arm during the median 2.8 years follow‐up period [6]. Similarly, the composite of stroke, systemic embolism, major bleeding, and death occurred in 16.8% of the patients in our study during the median 2‐year follow‐up.

Although RCTs provide foundational evidence for the assessment of the safety and efficacy of novel drugs in selected patients, real‐world observational studies offer insights into the safety, efficacy, prescribing patterns, and clinical outcomes of these drugs in a large‐scale population. The real‐world XANTUS study assessed the safety and efficacy of rivaroxaban in routine clinical practice. All‐cause death occurred in 118 patients (1.9 events per 100 patient‐years) within the study treatment period which was comparable with the ROCKET‐AF trial [4, 17]. During the 1‐year follow‐up period of the Edoxaban treatment in routine clinical practice for patients with non‐valvular atrial fibrillation (ETNA‐AF) Europe study, all‐cause death occurred in 422 patients (3.5%/year) [18]. At the 2‐year of ETNA‐AF Europe study, 937 patients died (incidence proportion: 7.1%) [19]. In the present study, the all‐cause mortality rate during the median 2‐years of follow‐up period was found to be 12.0% which was significantly higher than the previous real‐world studies [17, 18, 19]. The timing of the follow‐up period may be an influencing factor in the results of our study, particularly in the all‐cause mortality rate. The follow‐up period of this study and the global outbreak of coronavirus disease 2019 (COVID‐19) coincided with the same time. Several reports showed a high prevalence of cardiovascular diseases in patients with COVID‐19 and higher mortality during the COVID‐19 than before the pandemic [20, 21]. Beyond the direct effects of infection, indirect effects comprising reductions in rates of referrals, diagnosis, and treatment may have fatal long‐term consequences [20]. As an example, It is well‐known that the use of anticoagulant treatments reduces all‐cause mortality and pulmonary embolism in patients with COVID‐19, however, a reduction in the utilization of physician‐prescribed pharmaceuticals, such as oral anticoagulants, during the COVID‐19 lockdown has been reported [22, 23].

At present, there is a lack of data available for the guidance of selection between factor Xa inhibitors. A direct comparison between rivaroxaban, apixaban, and edoxaban for safety and efficacy in patients with AF was not conducted within a RCT. For this purpose, Talmor‐Barkan et al. [9] conducted an observational study to emulate a target trial for head‐to‐head comparison of DOACs therapy (rivaroxaban, apixaban, and dabigatran) by propensity score matching analysis. Six‐year follow‐up results of 56,553 patients showed that the all‐cause mortality and ischemic stroke rates were significantly lower in patients treated with rivaroxaban than those treated with apixaban. Furthermore, the overall bleeding rates were comparable between rivaroxaban, apixaban, and dabigatran. The findings of our study indicate that the rate of net clinical outcomes, the incidence of ischaemic stroke, and the all‐cause mortality rate were significantly lower in the rivaroxaban group than in the apixaban and edoxaban groups. The incidence of major bleeding was comparable among the three factor Xa inhibitors. These results are consistent with those of the aforementioned study from Israel. However, there is a critical point that needs to be emphasized at this point. A key limitation of our study is that, in contrast to the approach taken by Talmor‐Barkan et al. [9], we did not undertake a direct, head‐to‐head comparison of factor Xa inhibitors. In our study, there are differences in the baseline characteristics of the patients in these treatment groups. Such differences may potentially have affected the clinical outcomes. For instance, patients receiving apixaban therapy are older and have higher CHA_2_DS_2_‐VASc scores than those receiving rivaroxaban or edoxaban therapy. To illustrate further, the proportion of patients with a history of ischaemic stroke or systemic embolism and a history of intracranial hemorrhage was higher in the edoxaban group than in the rivaroxaban and apixaban groups. As evidenced by these examples, notable differences in the baseline characteristics of the patients may have been a contributing factor to the favorable outcomes observed with rivoraxaban.

Several clinical studies have demonstrated a higher risk of stroke in women compared to men in patients with AF [24, 25]. Therefore, female sex was incorporated into a number of stroke risk stratification models, including the CHA_2_DS_2_‐VASc score [1, 7]. However, we did not find an association between female gender and the occurrence of stroke in our study. Moreover, we showed an independent association between the male gender and the development of net clinical outcomes. This result gives rise to a matter of great importance: Does the female gender truly constitute a risk factor? Subgroup analysis of the Losartan Intervention for Endpoint (LIFE) study demonstrated that females with a history of AF did not have a higher stroke risk than males [26]. An analysis of the Danish National Patient Registry including a total of 239,671 individuals revealed that female sex is a risk modifier rather than a risk factor for stroke in patients with atrial fibrillation [27]. Similar to our results, some previous observational studies showed that the male gender increases the risk of clinical outcomes in patients with AF [28, 29]. These results suggest that the impact of gender on clinical outcomes in patients with AF is inconclusive. This highlights the necessity for future randomized studies on this subject.

The prescription of reduced doses of DOACs, particularly apixaban, is more frequent in clinical practice than in RCTs [13, 30, 31]. For instance, apixaban was the most commonly underdosed DOAC in the ORBIT‐AF II Registry [32]. The prescription of a reduced dose of apixaban varies between 15.5% and 25.1% in clinical practice, while this was only 4.6% in the ARISTOTLE trial [5, 13, 30, 31]. Regarding rivaroxaban, 17% to 21% of the patients received a reduced dose in a real‐life setting which is more comparable to 20.7% of the patients enrolled in ROCKET‐AF [4, 13, 30, 31]. Although it is anticipated that underdosing (inappropriate low dose) of factor Xa inhibitors will result in an elevated risk of stroke and other ischemic events, or overdosing (inappropriate high dose) is expected to increase the risk of bleeding events, the results of observational studies are conflicting [33]. In the present study, we found that the prescription of inappropriate low or high doses of factor Xa inhibitors, except the usage of apixaban 2.5 mg B.I.D., was not independently associated with the net clinical outcomes among the entire study population. The usage of apixaban 2.5 mg B.I.D was independently associated with the occurrence of both net clinical and efficacy outcomes. The results of our study are comparable to those reported by Yao et al. [34], who found that patients treated with apixaban 2.5 mg B.I.D. had an increased risk of stroke, whereas there were no significant differences in the risk of stroke in patients treated with reduced doses of rivaroxaban or dabigatran. Another observational study demonstrated that thromboembolic events occurred in 10.7% of patients treated with a reduced dose of apixaban which is significantly higher than in patients treated with reduced doses of rivaroxaban (3.6%) and dabigatran (5.1%) [35]. This may be associated with the pharmacodynamic and pharmacokinetic characteristics of apixaban. Previous studies reported that patients treated with the reduced dose of rivaroxaban had similar rivaroxaban concentrations in trough samples to patients treated with the standard dose of rivaroxaban and there was no significant intra‐individual variability for trough or peak rivaroxaban concentrations [36]. Similarly, patients receiving dabigatran 110 mg B.I.D. had similar dabigatran concentrations in both trough and peak samples compared to patients receiving dabigatran 150 mg B.I.D. treatment [37]. However, the prescription of a reduced dose of apixaban is associated with 50% lower plasma concentrations in patients with normal renal function and aged <75 years, and may be at risk of an undesirably low trough concentration if the dose reduction criteria are not strictly followed [38, 39]. These results suggest that the apixaban 2.5 mg treatment should be used in highly selected patients who had appropriate dose reduction criteria and these patients should be closely monitored, especially for the risk of ischemic events.

It is a well‐known entity that heart failure is associated with worse outcomes in patients with AF [40]. Similarly, we found that the presence of heart failure was an independent risk factor for the development of clinical outcomes in our cohort. In line with our findings, a history of heart failure was identified as an independent risk factor of clinical outcomes in a prospective cohort study [41]. Furthermore, a history of heart failure was the strongest predictor of all‐cause mortality in the ETNA‐AF study [18, 19]. These findings indicate that patients with AF should undergo assessment for the presence of overt or subclinical heart failure and emphasize the necessity of identifying optimal medical treatments for both clinical conditions.

Vulnerable older patients with AF who had low body weight, high bleeding risk, multiple comorbidities, or polypharmacy were underrepresented in the three pivotal trials [4, 5, 6]. These patients are at an elevated risk of mortality from any cause, as well as of stroke and bleeding [42]. A meta‐analysis of 1, 187, 000 individuals reported that frail AF patients were associated with a higher risk of all‐cause mortality, stroke, and bleeding when compared to robust individuals [43]. In keeping with these findings, we demonstrated older age, low body weight, high bleeding risk, and multiple comorbidities (including hypertension or chronic liver failure) as powerful indicators of adverse clinical outcomes in our analysis as well.

Our study has several limitations. First, the main limitation of our study was its observational and retrospective design, which may have resulted in biases in patient evaluation, selection, and attrition. Secondly, a direct, head‐to‐head comparison of factor Xa inhibitors was not undertaken, the unmeasured confounding factors and differences in the baseline characteristics of the study population may lead to overestimation or underestimation of treatment effects. Third, it should be noted that the study population was only enrolled in cardiology outpatient clinics in a single country. This population did not include those presenting at family medicine or internal medicine outpatient clinics. These factors may have an impact on the external validation of our study. Finally, the investigators themselves reported the primary and secondary study endpoints, and it remains possible that some variability may have been introduced during the reporting phase. Due to these limitations, the results of this study should be interpreted carefully.

## 5 Conclusion

The follow–up results of the ANATOLIA–AF study provide detailed data about the incidence and predictors of clinical outcomes in real‐life patients with AF treated with oral factor Xa inhibitors. Although the observed net clinical outcome rate appears to support the current evidence of factor Xa inhibitors in a real‐world clinical setting, the all‐cause mortality rate during the median 2‐year follow‐up period was higher than the previous reports. This may be particularly related to the excess mortality rates during the COVID‐19 pandemic. Our results showed that the rate of net clinical outcomes, the incidence of ischaemic stroke, and the all‐cause mortality rate were significantly lower in the rivaroxaban group than in the apixaban and edoxaban groups. However, it is important to consider that differences in patients' baseline characteristics may be a contributing factor to the favorable outcomes observed with rivaroxaban. These results underscore the necessity for future RCTs that will compare rivaroxaban, apixaban and edoxaban in order to more precisely inform the use of the different DOACs in clinical practice. The present study identified several clinical factors that are independently associated with the development of adverse clinical outcomes. These factors include older age, male sex, low body weight, high bleeding risk, the presence of hypertension, chronic heart failure, chronic liver failure, and apixaban 2.5 mg B.I.D. treatment. Our results highlight the need to optimize the management of patients with different comorbidities and AF, to reduce the risk of adverse clinical outcomes.

## Conflicts of Interest

The authors declare no conflict of interest.

## Supporting information

Supporting information.

Supporting information.

Supporting information.

Supporting information.

## Data Availability

The data that support the findings of this study are available from the corresponding author, upon reasonable request.
