# Supplementary figures and images for: Incidence and Predictors of Clinical Outcomes in Real‐Life Patients With Atrial Fibrillation Treated With Oral Factor Xa Inhibitors: The Follow‐Up Results of the ANATOLIA‐AF Study
Source: Clin Cardiol. 2025 Jan 27;48(1):e70088. doi: 10.1002/clc.70088 (PMC11773160; doi:10.1002/clc.70088)

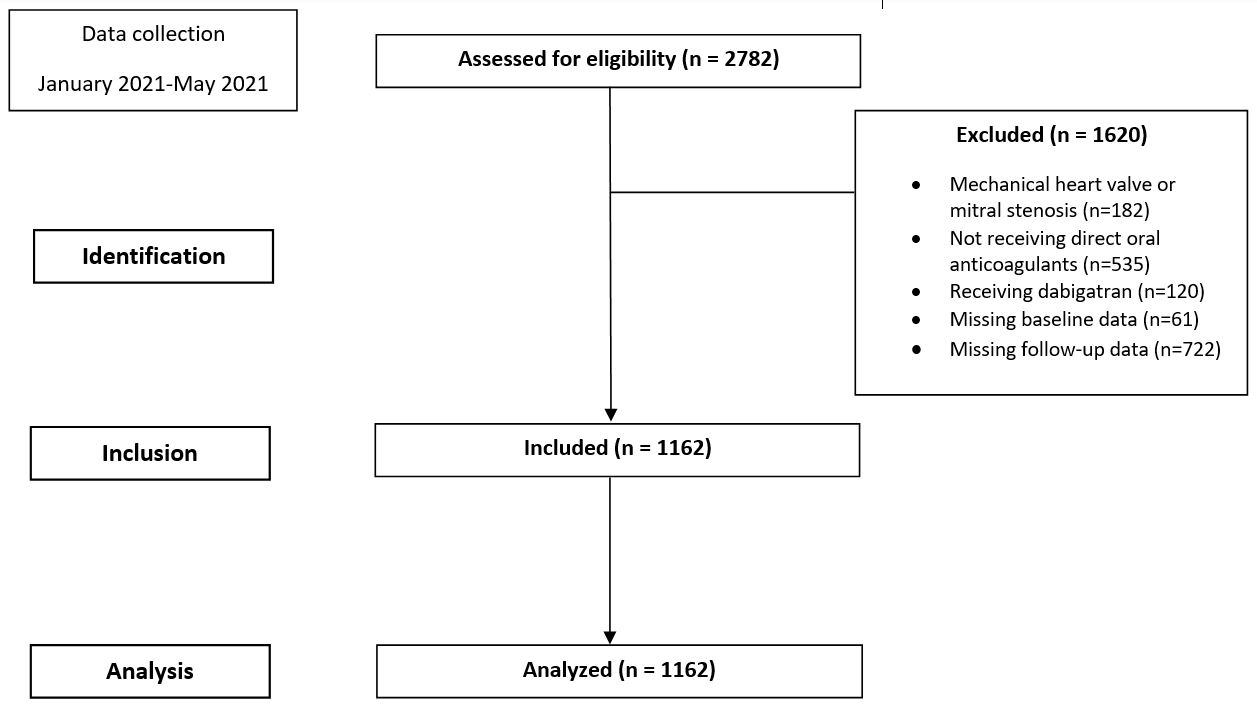

Supplement: Supplementary file 1 — Supporting information. [file CLC-48-e70088-s001.JPG]

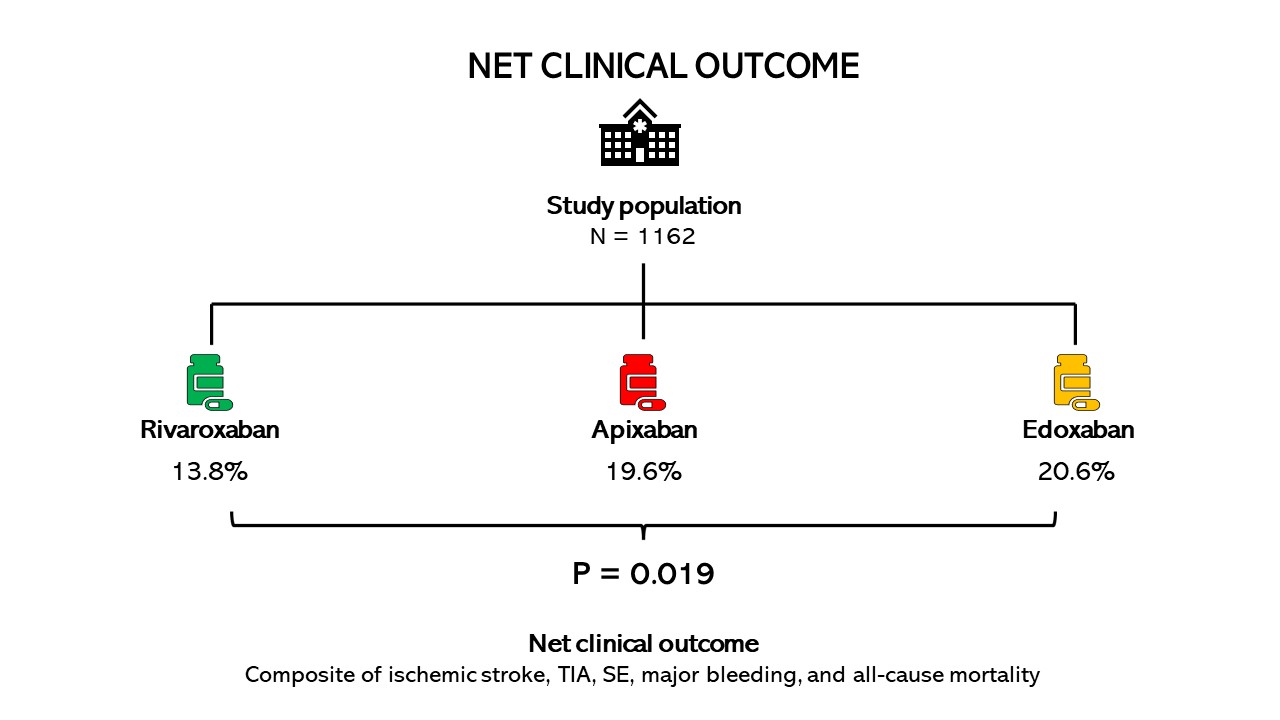

Supplement: Supplementary file 2 — Supporting information. [file CLC-48-e70088-s003.JPG]

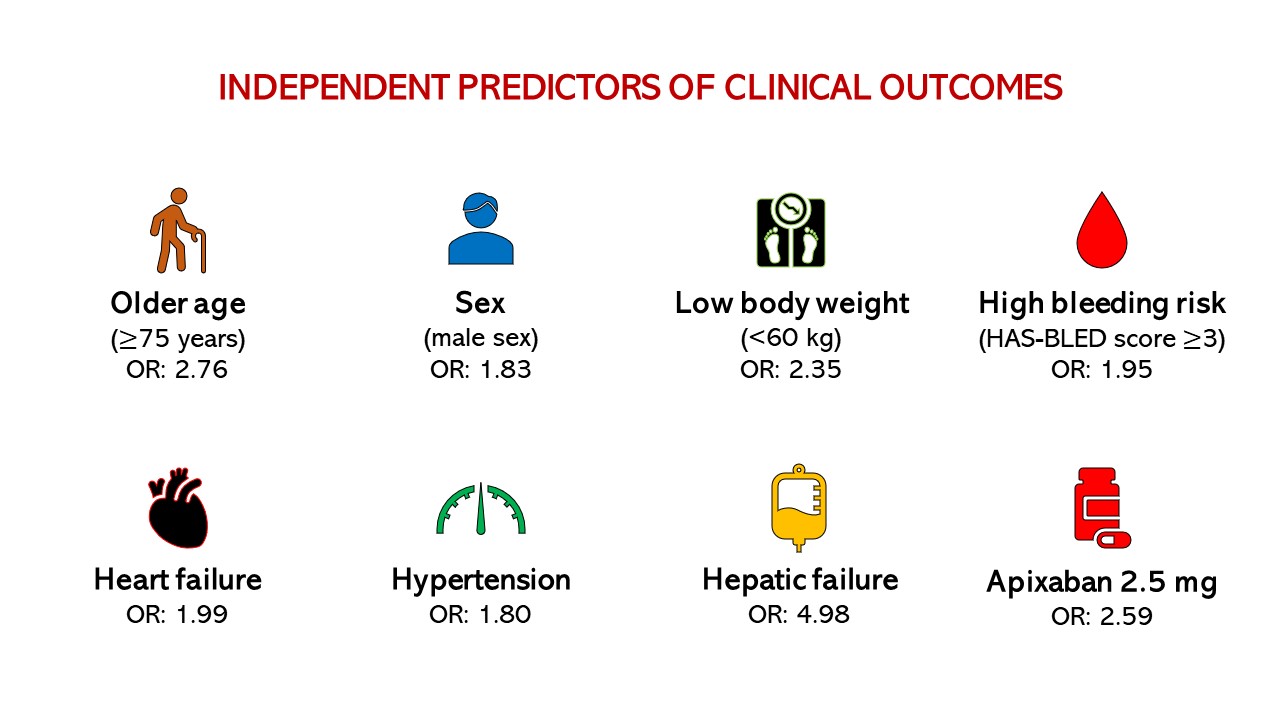

Supplement: Supplementary file 3 — Supporting information. [file CLC-48-e70088-s004.JPG]
