# Supplementary material for: Incidence and Predictors of Clinical Outcomes in Real‐Life Patients With Atrial Fibrillation Treated With Oral Factor Xa Inhibitors: The Follow‐Up Results of the ANATOLIA‐AF Study
Source: Clin Cardiol. 2025 Jan 27;48(1):e70088. doi: 10.1002/clc.70088 (PMC11773160; doi:10.1002/clc.70088)
Supplement: Supplementary file 4 — Supporting information. [file CLC-48-e70088-s002.docx]

**Supplementary table 1. Baseline demographic and clinical characteristics of the study population**

| **Variable** |  | **Total number of patients** |
| --- | --- | --- |
| **Demographics** |  |  |
| - Age, median (IQR and range), years | 72 (12 and 28–96) | 1162 |
| - Age group, n (%) |  | 1162 |
| - <65 years | 244 (21.0) |  |
| - 65 – 74 years | 453 (39.0) |  |
| - ≥75 years | 465 (40.0) |  |
| - Female sex, n (%) | 693 (59.6) | 1162 |
| - BMI, median (IQR), kg/m^2^ | 28.0 (6.1) | 1161 |
| - Low body weight (≤60 kg), n (%) | 119 (10.2) | 1161 |
| **AF-related information** |  |  |
| - CHA_2_DS_2_-VASc score, median (IQR) | 4.0 (2.0) | 1162 |
| - CHA_2_DS_2_-VASc score, mean ± SD | 3.8 ± 1.5 | 1162 |
| - High stroke risk, n (%) | 1023 (88.1) | 1162 |
| - HAS-BLED score, median (IQR) | 1.0 (1.0) | 1162 |
| - HAS-BLED score, mean ± SD | 1.5 ± 1.0 | 1162 |
| - High bleeding risk, n (%) | 151 (13.0) | 1162 |
| **Medical history** |  |  |
| - Previous stroke and/or TIA, n (%) | 187 (16.1) | 1161 |
| - Coronary artery disease, n (%) | 394 (33.9) | 1162 |
| - Chronic heart failure, n (%) | 463 (39.9) | 1161 |
| - Hypertension, n (%) | 921 (79.3) | 1161 |
| - Diabetes mellitus, n (%) | 361 (31.1) | 1161 |
| - Chronic kidney disease, n (%) | 378 (32.6) | 1161 |
| - Chronic liver failure, n (%) | 10 (0.9) | 1162 |
| - Anemia, n (%) | 416 (35.9) | 1160 |
| - Concomitant antiplatelet therapy, n (%) | 122 (10.5) | 1161 |
| **Previous bleeding history** |  |  |
| - Major bleeding, n (%) | 36 (3.1) | 1162 |
| - CRNM bleeding, n (%) | 56 (4.8) | 1162 |
| - Major and/or CRNM bleeding, n (%) | 88 (7.6) | 1162 |
| - Minor bleeding, n (%) | 287 (24.7) | 1162 |
| - History of GI bleeding, n (%) | 69 (6.0) | 1142 |
| - History of ICH, n (%) | 8 (0.7) | 1138 |
| **Factor Xa inhibitors** |  |  |
| - Rivaroxaban, n (%) | 589 (50.7) | 1162 |
| - Apixaban, n (%) | 403 (34.7) | 1162 |
| - Edoxaban, n (%) | 170 (14.6) | 1162 |
| - Appropriate reduced dose, n (%) | 116 (10.0) | 1161 |
| - Appropriate standard dose, n (%) | 804 (69.3) | 1161 |
| - Inappropriate reduced dose, n (%) | 176 (15.2) | 1161 |
| - Inappropriate standard dose, n (%) | 65 (5.6) | 1161 |
| **Laboratory data** |  |  |
| - Serum creatinine, mean ± SD, mg/dL | 1.00 ± 0.39 | 1162 |
| - GFR, mean ± SD, mg/dL | 71 ± 24 | 1161 |
| - GFR group, n (%) |  | 1161 |
| - GFR ≥60 mL/min/1.73 m^2^ | 783 (67.4) |  |
| - GFR 30 – 59 mL/min/1.73 m^2^ | 339 (29.2) |  |
| - GFR 15 – 29 mL/min/1.73 m^2^ | 39 (3.4) |  |
| - Hemoglobin, mean ± SD, mg/dL | 12.9 ± 2.0 | 1160 |
| - Platelet, median (IQR), mL/min | 228,000 (87,000) | 1160 |
| IQR = interquartile range; SD = standart deviation; BMI = body mass index; AF = atrial fibrillation; CHA2DS2-VASc = congestive heart failure, hypertension, age ≥75 (2 points), diabetes, stroke (2 points), vascular disease, age 65-74, sex category (female); HAS-BLED = uncontrolled hypertension, abnormal renal and liver function (1 point each), stroke, bleeding, labile international normalized ratios, elderly (age >65 years), drugs or alcohol (1 point each) (concomitant use of antiplatelet agents or non-steroidal anti-inflammatory drugs, alcohol abuse); TIA = transient ischemic attack; CRNM = clinically relevant non-major bleeding; GI = gastrointestinal; ICH = intracranial hemorrhage; GFR = glomerular filtration rate. | | |

**Supplementary table 2. Baseline demographic and clinical characteristics of the study population according to the factor Xa inhibitors**

| **Variable** | **Rivaroxaban** | **Apixaban** | **Edoxaban** | ***P* – value** |
| --- | --- | --- | --- | --- |
| **Demographics** |  |  |  |  |
| - Age, median (IQR), years | 71 (13) | 73 (12) | 71.5 (16) | 0.005 |
| - Age group, n (%) |  |  |  |  |
| - <65 years | 135 (22.9) | 62 (15.4) | 47 (27.6) | 0.001 |
| - 65 – 74 years | 240 (40.7) | 161 (40.0) | 52 (30.6) |  |
| - ≥75 years | 214 (36.3) | 180 (44.7) | 71 (41.8) |  |
| - Female sex, n (%) | 342 (58.1) | 258 (64.0) | 93 (54.7) | 0.063 |
| - BMI, median (IQR), kg/m^2^ | 28.1 (6.1) | 28.0 (6.2) | 27.9 (6.1) | 0.72 |
| - Low body weight (≤60 kg), n (%) | 59 (10.0) | 42 (10.4) | 18 (10.6) | 0.96 |
| **AF-related information** |  |  |  |  |
| - CHA_2_DS_2_-VASc score, mean ± SD | 3.67 ± 1.53 | 4.07 ± 1.47 | 3.70 ± 1.65 | <0.001 |
| - High stroke risk, n (%) | 507 (86.1) | 371 (92.1) | 145 (85.3) | 0.008 |
| - HAS-BLED score, mean ± SD | 1.43 ± 0.91 | 1.59 ± 0.98 | 1.48 ± 0.96 | 0.053 |
| - High bleeding risk, n (%) | 69 (11.7) | 61 (15.1) | 21 (12.4) | 0.27 |
| **Medical history** |  |  |  |  |
| - Previous stroke and/or TIA, n (%) | 78 (13.2) | 74 (18.4) | 35 (20.7) | 0.021 |
| - Coronary artery disease, n (%) | 189 (32.1) | 144 (35.7) | 61 (35.9) | 0.41 |
| - Chronic heart failure, n (%) | 229 (38.9) | 156 (38.7) | 78 (45.9) | 0.22 |
| - Hypertension, n (%) | 460 (78.1) | 336 (83.6) | 125 (73.5) | 0.015 |
| - Diabetes mellitus, n (%) | 182 (30.9) | 133 (33.1) | 46 (27.1) | 0.36 |
| - Chronic kidney disease, n (%) | 174 (29.5) | 151 (37.6) | 53 (31.2) | 0.028 |
| - Chronic liver failure, n (%) | 4 (0.7) | 4 (1.0) | 2 (1.2) | 0.77 |
| - Anemia, n (%) | 206 (35.0) | 156 (38.8) | 54 (31.8) | 0.23 |
| - Concomitant antiplatelet therapy, n (%) | 62 (10.5) | 40 (9.9) | 20 (11.8) | 0.80 |
| **Previous bleeding history** |  |  |  |  |
| - Major bleeding, n (%) | 20 (3.4) | 13 (3.2) | 3 (1.8) | 0.54 |
| - CRNM bleeding, n (%) | 24 (4.1) | 22 (5.5) | 10 (5.9) | 0.47 |
| - Major and/or CRNM bleeding, n (%) | 42 (7.1) | 33 (8.2) | 13 (7.6) | 0.82 |
| - Minor bleeding, n (%) | 140 (23.8) | 108 (26.8) | 39 (22.9) | 0.47 |
| - History of GI bleeding, n (%) | 33 (5.7) | 27 (6.8) | 9 (5.4) | 0.70 |
| - History of ICH, n (%) | 2 (0.3) | 2 (0.5) | 4 (2.4) | 0.017 |
| **Appropriate or inappropriate dosages** |  |  |  |  |
| - Appropriate reduced dose, n (%) | 69 (11.7) | 28 (7.0) | 19 (11.2) | 0.043 |
| - Appropriate standard dose, n (%) | 393 (66.7) | 297 (73.9) | 114 (67.1) | 0.045 |
| - Inappropriate reduced dose, n (%) | 95 (16.1) | 68 (16.7) | 13 (7.6) | 0.012 |
| - Inappropriate standard dose, n (%) | 32 (5.4) | 9 (2.2) | 24 (14.1) | <0.001 |
| **Laboratory data** |  |  |  |  |
| - Serum creatinine, mean ± SD, mg/dL | 0.98 ± 0.39 | 1.02 ± 0.40 | 1.00 ± 0.31 | 0.40 |
| - GFR, mean ± SD, mg/dL | 72 ± 23 | 69 ± 25 | 72 ± 24 | 0.10 |
| - GFR group, n (%) |  |  |  |  |
| - GFR ≥60 mL/min/1.73 m^2^ | 415 (70.4) | 251 (62.4) | 117 (68.8) | 0.070 |
| - GFR 30 – 59 mL/min/1.73 m^2^ | 159 (27.0) | 132 (32.9) | 48 (28.2) |  |
| - GFR 15 – 29 mL/min/1.73 m^2^ | 15 (2.6) | 19 (4.7) | 5 (3.0) |  |
| - Hemoglobin, mean ± SD, mg/dL | 12.9 ± 2.1 | 12.6 ± 1.9 | 13.1 ± 2.1 | 0.007 |
| - Platelet, median (IQR), mL/min | 230,000 (86,000) | 227,000 (87,000) | 227,000 (85,000) | 0.86 |
| IQR = interquartile range; SD = standart deviation; BMI = body mass index; AF = atrial fibrillation; CHA2DS2-VASc = congestive heart failure, hypertension, age ≥75 (2 points), diabetes, stroke (2 points), vascular disease, age 65-74, sex category (female); HAS-BLED = uncontrolled hypertension, abnormal renal and liver function (1 point each), stroke, bleeding, labile international normalized ratios, elderly (age >65 years), drugs or alcohol (1 point each) (concomitant use of antiplatelet agents or non-steroidal anti-inflammatory drugs, alcohol abuse); TIA = transient ischemic attack; CRNM = clinically relevant non-major bleeding; GI = gastrointestinal; ICH = intracranial hemorrhage; DOAC = direct oral anticoagulants; GFR = glomerular filtration rate. | | | | |

**Supplementary table 3. Baseline demographic and clinical characteristics of patients without and with clinical outcomes^#^**

| **Variable** | **Patients without clinical outcomes** | **Patients with clinical outcomes** | ***P* – value** |
| --- | --- | --- | --- |
| **Demographics** |  |  |  |
| - Age, median (IQR), years | 71 (13) | 77 (12) | <0.001 |
| - Age group, n (%) |  |  |  |
| - <65 years | 224 (91.8) | 20 (8.2) | <0.001 |
| - 65 – 74 years | 390 (86.1) | 63 (13.9) |  |
| - ≥75 years | 353 (75.9) | 112 (24.1) |  |
| - Sex, n (%) |  |  |  |
| - Female | 590 (85.1) | 103 (14.9) | 0.033 |
| - Male | 377 (80.4) | 92 (19.6) |  |
| - BMI, median (IQR), kg/m^2^ | 28.2 (5.9) | 27.3 (6.5) | 0.027 |
| - Body weight, n (%) |  |  |  |
| - Low body weight (≤60 kg) | 83 (69.7) | 36 (30.3) | <0.001 |
| - High body weight (>60 kg) | 883 (84.7) | 159 (15.3) |  |
| **AF-related information** |  |  |  |
| - CHA_2_DS_2_-VASc score, mean ± SD | 3.68 ± 1.52 | 4.45 ± 1.45 | <0.001 |
| - Stroke risk, n (%) |  |  |  |
| - Low stroke risk* | 128 (92.1) | 11 (7.9) | 0.003 |
| - High stroke risk* | 839 (82.0) | 184 (18.0) |  |
| - HAS-BLED score, mean ± SD | 1.40 ± 0.92 | 1.93 ± 0.95 | <0.001 |
| - Bleeding risk, n (%) |  |  |  |
| - Low bleeding risk** | 868 (85.9) | 143 (14.1) | <0.001 |
| - High bleeding risk** | 99 (65.6) | 52 (34.4) |  |
| **Medical history** |  |  |  |
| - Previous stroke and/or TIA, n (%) |  |  |  |
| - No | 824 (84.6) | 150 (15.4) | 0.004 |
| - Yes | 142 (75.9) | 45 (24.1) |  |
| - Coronary artery disease, n (%) |  |  |  |
| - No | 647 (84.2) | 121 (15.8) | 0.19 |
| - Yes | 320 (81.2) | 74 (18.8) |  |
| - Chronic heart failure, n (%) |  |  |  |
| - No | 612 (87.7) | 86 (12.3) | <0.001 |
| - Yes | 354 (76.5) | 109 (23.5) |  |
| - Hypertension, n (%) |  |  |  |
| - No | 212 (88.3) | 28 (11.7) | 0.017 |
| - Yes | 754 (81.9) | 167 (18.1) |  |
| - Diabetes mellitus, n (%) |  |  |  |
| - No | 669 (83.6) | 131 (16.4) | 0.56 |
| - Yes | 297 (82.3) | 64 (17.7) |  |
| - Chronic kidney disease, n (%) |  |  |  |
| - No | 681 (87.0) | 102 (13.0) | <0.001 |
| - Yes | 285 (75.4) | 93 (24.6) |  |
| - Chronic liver failure, n (%) |  |  |  |
| - No | 962 (83.5) | 190 (16.5) | 0.005 |
| - Yes | 5 (50.0) | 5 (50.0) |  |
| - Anemia, n (%) |  |  |  |
| - No | 648 (87.1) | 96 (12.9) | <0.001 |
| - Yes | 318 (76.4) | 98 (23.6) |  |
| - Concomitant antiplatelet therapy, n (%) |  |  |  |
| - No | 867 (83.4) | 172 (16.6) | 0.52 |
| - Yes | 99 (81.1) | 23 (18.9) |  |
| **Previous bleeding history** |  |  |  |
| - Major bleeding, n (%) |  |  |  |
| - No | 946 (84.0) | 180 (16.0) | <0.001 |
| - Yes | 21 (58.3) | 15 (41.7) |  |
| - CRNM bleeding, n (%) |  |  |  |
| - No | 925 (83.6) | 181 (16.4) | 0.092 |
| - Yes | 42 (75.0) | 14 (25.0) |  |
| - Major and/or CRNM bleeding, n (%) |  |  |  |
| - No | 906 (84.4) | 168 (15.6) | <0.001 |
| - Yes | 61 (69.3) | 27 (30.7) |  |
| - Minor bleeding, n (%) |  |  |  |
| - No | 739 (84.5) | 136 (15.5) | 0.049 |
| - Yes | 228 (79.4) | 59 (20.6) |  |
| - History of GI bleeding, n (%) |  |  |  |
| - No | 908 (84.6) | 165 (15.4) | <0.001 |
| - Yes | 47 (68.1) | 22 (31.9) |  |
| - History of ICH, n (%) |  |  |  |
| - No | 945 (83.6) | 185 (16.4) | 0.51 |
| - Yes | 6 (75.0) | 2 (25.0) |  |
| **DOAC type** |  |  |  |
| - Rivaroxaban, n (%) | 508 (86.2) | 81 (13.8) | 0.019 |
| - Apixaban, n (%) | 324 (80.4) | 79 (19.6) |  |
| - Edoxaban, n (%) | 135 (79.4) | 35 (20.6) |  |
| **DOAC dosages** |  |  |  |
| - Rivaroxaban 20 mg OD, n (%) | 379 (89.2) | 46 (10.8) | <0.001 |
| - Rivaroxaban 15 mg OD, n (%) | 129 (78.7) | 35 (21.3) |  |
| - Apixaban 5 mg BID, n (%) | 263 (85.7) | 44 (14.3) |  |
| - Apixaban 2.5 mg BID, n (%) | 61 (63.5) | 35 (36.5) |  |
| - Edoxaban 60 mg OD, n (%) | 113 (81.9) | 25 (18.1) |  |
| - Edoxaban 30 mg OD, n (%) | 22 (68.8) | 10 (31.3) |  |
| **Appropriate or inappropriate dosages** |  |  |  |
| - Appropriate reduced dose, n (%) | 78 (67.2) | 38 (32.3) | <0.001 |
| - Appropriate standard dose, n (%) | 705 (87.7) | 99 (12.3) |  |
| - Inappropriate reduced dose, n (%) | 134 (87.7) | 42 (23.9) |  |
| - Inappropriate standard dose, n (%) | 49 (75.4) | 16 (24.6) |  |
| **Laboratory data** |  |  |  |
| - Serum creatinine, mean ± SD, mg/dL | 0.97 ± 0.32 | 1.16 ± 0.59 | <0.001 |
| - GFR, mean ± SD, mg/dL | 73 ± 24 | 63 ± 25 | <0.001 |
| - GFR group, n (%) |  |  |  |
| - GFR ≥60 mL/min/1.73 m^2^ | 681 (87.0) | 102 (13.0) | <0.001 |
| - GFR 30 – 59 mL/min/1.73 m^2^ | 261 (77.0) | 78 (23.0) |  |
| - GFR 15 – 29 mL/min/1.73 m^2^ | 24 (66.7) | 12 (33.3) |  |
| - Hemoglobin, mean ± SD, mg/dL | 13.0 ± 1.9 | 12.3 ± 2.3 | <0.001 |
| - Platelet, median (IQR), mL/min | 230,000 (85,000) | 223,000 (100,000) | 0.25 |
| #Clinical outcomes (=net clinical outcome): Composite of ischemic stroke, TIA, systemic embolism, major bleeding, and/or all-cause mortality.  *Low stroke risk = CHA_2_DS_2_-VASc score <3 (female) and CHA_2_DS_2_-VASc score <2 (male); and high stroke risk = CHA_2_DS_2_-VASc score ≥3 (female) and CHA_2_DS_2_-VASc score ≥2 (male).  *Low bleeding risk = HAS-BLED score <3; and high bleeding risk = HAS-BLED ≥3.  IQR = interquartile range; BMI = body mass index; CHA2DS2-VASc = congestive heart failure, hypertension, age ≥75 (2 points), diabetes, stroke (2 points), vascular disease, age 65-74, sex category (female); HAS-BLED = uncontrolled hypertension, abnormal renal and liver function (1 point each), stroke, bleeding, labile international normalized ratios, elderly (age >65 years), drugs or alcohol (1 point each) (concomitant use of antiplatelet agents or non-steroidal anti-inflammatory drugs, alcohol abuse); TIA = transient ischemic attack; CRNM = clinically relevant non-major bleeding; GI = gastrointestinal; ICH = intracranial hemorrhage; DOAC = direct oral anticoagulant; SD = standard deviation; GFR = glomerular filtration rate. | | | |

**Supplementary table 4. Baseline demographic and clinical characteristics of patients without and with safety outcomes^#^**

| **Variable** | **Patients without safety outcomes** | **Patients with safety outcomes** | ***P* – value** |
| --- | --- | --- | --- |
| **Demographics** |  |  |  |
| - Age, median (IQR), years | 72 (13) | 75.5 (15) | 0.014 |
| - Age group, n (%) |  |  |  |
| - <65 years | 226 (92.6) | 18 (7.4) | 0.035 |
| - 65 – 74 years | 426 (94.0) | 27 (6.0) |  |
| - ≥75 years | 416 (89.5) | 49 (10.5) |  |
| - Sex, n (%) |  |  |  |
| - Female | 640 (92.4) | 53 (7.6) | 0.50 |
| - Male | 428 (91.3) | 41 (8.7) |  |
| - BMI, median (IQR), kg/m^2^ | 28.1 (6.1) | 27.2 (5.5) | 0.13 |
| - Body weight, n (%) |  |  |  |
| - Low body weight (≤60 kg) | 104 (87.4) | 15 (12.6) | 0.057 |
| - High body weight (>60 kg) | 963 (92.4) | 79 (7.6) |  |
| **AF-related information** |  |  |  |
| - CHA_2_DS_2_-VASc score, mean ± SD | 3.79 ± 1.53 | 4.12 ± 1.61 | 0.029 |
| - Stroke risk, n (%) |  |  |  |
| - Low stroke risk* | 130 (93.5) | 9 (6.5) | 0.45 |
| - High stroke risk* | 938 (91.7) | 85 (8.3) |  |
| - HAS-BLED score, mean ± SD | 1.46 ± 0.94 | 1.86 ± 1.00 | <0.001 |
| - Bleeding risk, n (%) |  |  |  |
| - Low bleeding risk** | 942 (93.2) | 69 (6.8) | <0.001 |
| - High bleeding risk** | 126 (83.4) | 25 (16.6) |  |
| **Medical history** |  |  |  |
| - Previous stroke and/or TIA, n (%) |  |  |  |
| - No | 897 (92.1) | 77 (7.9) | 0.58 |
| - Yes | 170 (90.9) | 17 (9.1) |  |
| - Coronary artery disease, n (%) |  |  |  |
| - No | 703 (91.5) | 65 (8.5) | 0.51 |
| - Yes | 365 (92.6) | 29 (7.4) |  |
| - Chronic heart failure, n (%) |  |  |  |
| - No | 645 (92.4) | 53 (7.6) | 0.44 |
| - Yes | 422 (91.1) | 41 (8.9) |  |
| - Hypertension, n (%) |  |  |  |
| - No | 229 (95.4) | 11 (4.6) | 0.025 |
| - Yes | 838 (91.0) | 83 (9.0) |  |
| - Diabetes mellitus, n (%) |  |  |  |
| - No | 733 (91.6) | 67 (8.4) | 0.60 |
| - Yes | 334 (92.5) | 27 (7.5) |  |
| - Chronic kidney disease, n (%) |  |  |  |
| - No | 722 (92.2) | 61 (7.8) | 0.58 |
| - Yes | 345 (91.3) | 33 (8.7) |  |
| - Chronic liver failure, n (%) |  |  |  |
| - No | 1058 (91.8) | 94 (8.2) | 0.34 |
| - Yes | 10 (100.0) | 0 (0.0) |  |
| - Anemia, n (%) |  |  |  |
| - No | 702 (94.4) | 42 (5.6) | <0.001 |
| - Yes | 365 (87.7) | 51 (12.3) |  |
| - Concomitant antiplatelet therapy, n (%) |  |  |  |
| - No | 953 (91.7) | 86 (8.3) | 0.51 |
| - Yes | 114 (93.4) | 8 (6.6) |  |
| **Previous bleeding history** |  |  |  |
| - Major bleeding, n (%) |  |  |  |
| - No | 1043 (92.6) | 83 (7.4) | <0.001 |
| - Yes | 25 (69.4) | 11 (30.6) |  |
| - CRNM bleeding, n (%) |  |  |  |
| - No | 1022 (92.4) | 84 (7.6) | 0.006 |
| - Yes | 46 (82.1) | 10 (17.9) |  |
| - Major and/or CRNM bleeding, n (%) |  |  |  |
| - No | 1000 (93.1) | 74 (6.9) | <0.001 |
| - Yes | 68 (77.3) | 20 (22.7) |  |
| - Minor bleeding, n (%) |  |  |  |
| - No | 820 (93.7) | 55 (6.3) | <0.001 |
| - Yes | 248 (86.4) | 39 (13.6) |  |
| - History of GI bleeding, n (%) |  |  |  |
| - No | 998 (93.0) | 75 (7.0) | <0.001 |
| - Yes | 54 (78.3) | 15 (21.7) |  |
| - History of ICH, n (%) |  |  |  |
| - No | 1041 (92.1) | 89 (7.9) | 0.62 |
| - Yes | 7 (87.5) | 1 (12.5) |  |
| **DOAC type** |  |  |  |
| - Rivaroxaban, n (%) | 543 (92.2) | 46 (7.8) | 0.90 |
| - Apixaban, n (%) | 370 (91.8) | 33 (8.2) |  |
| - Edoxaban, n (%) | 155 (91.2) | 15 (8.8) |  |
| **DOAC dosages** |  |  |  |
| - Rivaroxaban 20 mg OD, n (%) | 395 (92.9) | 30 (7.1) | 0.90 |
| - Rivaroxaban 15 mg OD, n (%) | 148 (90.2) | 16 (9.8) |  |
| - Apixaban 5 mg BID, n (%) | 283 (92.2) | 24 (7.8) |  |
| - Apixaban 2.5 mg BID, n (%) | 87 (90.6) | 9 (9.4) |  |
| - Edoxaban 60 mg OD, n (%) | 126 (91.3) | 12 (8.7) |  |
| - Edoxaban 30 mg OD, n (%) | 29 (90.6) | 3 (9.4) |  |
| **Appropriate or inappropriate dosages** |  |  |  |
| - Appropriate reduced dose, n (%) | 105 (90.5) | 11 (9.5) | 0.76 |
| - Appropriate standard dose, n (%) | 743 (92.4) | 61 (7.6) |  |
| - Inappropriate reduced dose, n (%) | 159 (90.3) | 17 (9.7) |  |
| - Inappropriate standard dose, n (%) | 60 (92.3) | 5 (7.7) |  |
| **Laboratory data** |  |  |  |
| - Serum creatinine, mean ± SD, mg/dL | 1.00 ± 0.39 | 1.01 ± 0.37 | 0.88 |
| - GFR, mean ± SD, mg/dL | 71 ± 24 | 70 ± 23 | 0.60 |
| - GFR group, n (%) |  |  |  |
| - GFR ≥60 mL/min/1.73 m^2^ | 722 (92.2) | 61 (7.8) | 0.83 |
| - GFR 30 – 59 mL/min/1.73 m^2^ | 309 (91.2) | 30 (8.8) |  |
| - GFR 15 – 29 mL/min/1.73 m^2^ | 33 (91.7) | 3 (8.3) |  |
| - Hemoglobin, mean ± SD, mg/dL | 13.0 ± 1.9 | 11.8 ± 2.6 | <0.001 |
| - Platelet, median (IQR), mL/min | 230,000 (87,000) | 221 (84,500) | 0.49 |
| #Safety outcomes: Composite of major bleeding and/or clinically relevant non-major bleeding.  *Low stroke risk = CHA_2_DS_2_-VASc score <3 (female) and CHA_2_DS_2_-VASc score <2 (male); and high stroke risk = CHA_2_DS_2_-VASc score ≥3 (female) and CHA_2_DS_2_-VASc score ≥2 (male).  *Low bleeding risk = HAS-BLED score <3; and high bleeding risk = HAS-BLED ≥3.  IQR = interquartile range; BMI = body mass index; CHA2DS2-VASc = congestive heart failure, hypertension, age ≥75 (2 points), diabetes, stroke (2 points), vascular disease, age 65-74, sex category (female); HAS-BLED = uncontrolled hypertension, abnormal renal and liver function (1 point each), stroke, bleeding, labile international normalized ratios, elderly (age >65 years), drugs or alcohol (1 point each) (concomitant use of antiplatelet agents or non-steroidal anti-inflammatory drugs, alcohol abuse); TIA = transient ischemic attack; CRNM = clinically relevant non-major bleeding; GI = gastrointestinal; ICH = intracranial hemorrhage; DOAC = direct oral anticoagulant; SD = standard deviation; GFR = glomerular filtration rate. | | | |

**Supplementary table 5. Baseline demographic and clinical characteristics of patients without and with efficacy outcomes^#^**

| **Variable** | **Patients without efficacy outcomes** | **Patients with efficacy outcomes** | ***P* – value** |
| --- | --- | --- | --- |
| **Demographics** |  |  |  |
| - Age, median (IQR), years | 72 (13) | 73 (10) | 0.38 |
| - Age group, n (%) |  |  |  |
| - <65 years | 239 (98.0) | 5 (2.0) | 0.22 |
| - 65 – 74 years | 432 (95.4) | 21 (4.6) |  |
| - ≥75 years | 448 (96.3) | 17 (3.7) |  |
| - Sex, n (%) |  |  |  |
| - Female | 663 (95.7) | 30 (4.3) | 0.16 |
| - Male | 456 (97.2) | 13 (2.8) |  |
| - BMI, median (IQR), kg/m^2^ | 28.7 (4.9) | 28.8 (5.4) | 0.82 |
| - Body weight, n (%) |  |  |  |
| - Low body weight (≤60 kg) | 113 (95.0) | 6 (5.0) | 0.41 |
| - High body weight (>60 kg) | 1005 (96.4) | 37 (3.6) |  |
| **AF-related information** |  |  |  |
| - CHA_2_DS_2_-VASc score, mean ± SD | 3.79 ± 1.54 | 4.34 ± 1.44 | 0.025 |
| - Stroke risk, n (%) |  |  |  |
| - Low stroke risk* | 137 (98.6) | 2 (1.4) | 0.13 |
| - High stroke risk* | 982 (96.0) | 41 (4.0) |  |
| - HAS-BLED score, mean ± SD | 1.47 ± 0.94 | 2.04 ± 0.95 | <0.001 |
| - Bleeding risk, n (%) |  |  |  |
| - Low bleeding risk** | 980 (96.9) | 31 (3.1) | 0.008 |
| - High bleeding risk** | 139 (92.1) | 12 (7.9) |  |
| **Medical history** |  |  |  |
| - Previous stroke and/or TIA, n (%) |  |  |  |
| - No | 948 (97.3) | 26 (2.7) | <0.001 |
| - Yes | 170 (90.9) | 17 (9.1) |  |
| - Coronary artery disease, n (%) |  |  |  |
| - No | 735 (95.7) | 33 (4.3) | 0.13 |
| - Yes | 384 (97.5) | 10 (2.5) |  |
| - Chronic heart failure, n (%) |  |  |  |
| - No | 674 (96.6) | 24 (3.4) | 0.55 |
| - Yes | 444 (95.6) | 19 (4.1) |  |
| - Hypertension, n (%) |  |  |  |
| - No | 232 (96.7) | 8 (3.3) | 0.73 |
| - Yes | 886 (96.2) | 35 (3.8) |  |
| - Diabetes mellitus, n (%) |  |  |  |
| - No | 766 (95.8) | 34 (4.3) | 0.14 |
| - Yes | 352 (97.5) | 9 (2.5) |  |
| - Chronic kidney disease, n (%) |  |  |  |
| - No | 751 (95.9) | 32 (4.1) | 0.32 |
| - Yes | 367 (97.1) | 11 (2.9) |  |
| - Chronic liver failure, n (%) |  |  |  |
| - No | 1109 (96.3) | 43 (3.7) | 0.53 |
| - Yes | 5 (100.0) | 0 (0.0) |  |
| - Anemia, n (%) |  |  |  |
| - No | 718 (96.5) | 26 (3.5) | 0.60 |
| - Yes | 399 (95.6) | 17 (4.1) |  |
| - Concomitant antiplatelet therapy, n (%) |  |  |  |
| - No | 1003 (96.5) | 36 (3.5) | 0.20 |
| - Yes | 115 (94.3) | 7 (5.7) |  |
| **Previous bleeding history** |  |  |  |
| - Major bleeding, n (%) |  |  |  |
| - No | 1084 (96.3) | 42 (3.7) | 0.76 |
| - Yes | 35 (97.2) | 1 (2.8) |  |
| - CRNM bleeding, n (%) |  |  |  |
| - No | 1067 (96.5) | 39 (3.5) | 0.16 |
| - Yes | 52 (92.9) | 4 (7.1) |  |
| - Major and/or CRNM bleeding, n (%) |  |  |  |
| - No | 1036 (96.5) | 38 (3.5) | 0.30 |
| - Yes | 83 (94.3) | 5 (5.7) |  |
| - Minor bleeding, n (%) |  |  |  |
| - No | 841 (96.1) | 34 (3.9) | 0.55 |
| - Yes | 278 (96.9) | 9 (3.1) |  |
| - History of GI bleeding, n (%) |  |  |  |
| - No | 1038 (96.7) | 35 (3.3) | 0.62 |
| - Yes | 66 (95.7) | 3 (4.3) |  |
| - History of ICH, n (%) |  |  |  |
| - No | 1092 (96.6) | 38 (3.4) | 0.59 |
| - Yes | 8 (100.0) | 0 (0.0) |  |
| **DOAC type** |  |  |  |
| - Rivaroxaban, n (%) | 576 (97.8) | 13 (2.2) | 0.014 |
| - Apixaban, n (%) | 384 (95.3) | 19 (4.7) |  |
| - Edoxaban, n (%) | 159 (93.5) | 11 (6.5) |  |
| **DOAC dosages** |  |  |  |
| - Rivaroxaban 20 mg OD, n (%) | 417 (98.1) | 8 (1.9) | <0.001 |
| - Rivaroxaban 15 mg OD, n (%) | 159 (97.0) | 5 (3.0) |  |
| - Apixaban 5 mg BID, n (%) | 297 (96.7) | 10 (3.3) |  |
| - Apixaban 2.5 mg BID, n (%) | 87 (90.6) | 9 (9.4) |  |
| - Edoxaban 60 mg OD, n (%) | 130 (94.2) | 8 (5.8) |  |
| - Edoxaban 30 mg OD, n (%) | 29 (90.6) | 3 (9.4) |  |
| **Appropriate or inappropriate dosages** |  |  |  |
| - Appropriate reduced dose, n (%) | 111 (95.7) | 5 (4.3) | 0.087 |
| - Appropriate standard dose, n (%) | 779 (96.9) | 25 (3.1) |  |
| - Inappropriate reduced dose, n (%) | 164 (93.2) | 12 (6.8) |  |
| - Inappropriate standard dose, n (%) | 64 (98.5) | 1 (1.5) |  |
| **Laboratory data** |  |  |  |
| - Serum creatinine, mean ± SD, mg/dL | 1.00 ± 0.38 | 1.02 ± 0.48 | 0.67 |
| - GFR, mean ± SD, mg/dL | 71 ± 24 | 73 ± 35 | 0.56 |
| - GFR group, n (%) |  |  |  |
| - GFR ≥60 mL/min/1.73 m^2^ | 751 (95.9) | 32 (4.1) | 0.48 |
| - GFR 30 – 59 mL/min/1.73 m^2^ | 330 (97.3) | 9 (2.7) |  |
| - GFR 15 – 29 mL/min/1.73 m^2^ | 35 (97.2) | 1 (2.8) |  |
| - Hemoglobin, mean ± SD, mg/dL | 12.9 ± 2.0 | 12.8 ± 1.9 | 0.77 |
| - Platelet, median (IQR), mL/min | 228,000 (86,000) | 234,000 (95,000) | 0.68 |
| #Efficacy outcomes: Composite of ischemic stroke and/or transient ischemic attack and/or systemic embolism.  *Low stroke risk = CHA_2_DS_2_-VASc score <3 (female) and CHA_2_DS_2_-VASc score <2 (male); and high stroke risk = CHA_2_DS_2_-VASc score ≥3 (female) and CHA_2_DS_2_-VASc score ≥2 (male).  *Low bleeding risk = HAS-BLED score <3; and high bleeding risk = HAS-BLED ≥3.  IQR = interquartile range; BMI = body mass index; CHA2DS2-VASc = congestive heart failure, hypertension, age ≥75 (2 points), diabetes, stroke (2 points), vascular disease, age 65-74, sex category (female); HAS-BLED = uncontrolled hypertension, abnormal renal and liver function (1 point each), stroke, bleeding, labile international normalized ratios, elderly (age >65 years), drugs or alcohol (1 point each) (concomitant use of antiplatelet agents or non-steroidal anti-inflammatory drugs, alcohol abuse); TIA = transient ischemic attack; CRNM = clinically relevant non-major bleeding; GI = gastrointestinal; ICH = intracranial hemorrhage; DOAC = direct oral anticoagulant; SD = standard deviation; GFR = glomerular filtration rate. | | | |

**Supplementary table 6. Univariate and multivariate analysis of the safety outcomes among study population^#^**

|  | **Univariable analysis** | | | **Multivariable analysis** | | | |
| --- | --- | --- | --- | --- | --- | --- | --- |
| **Variable** | **Odds ratio** | **95% CI** | **P – value** | **Odds ratio** | **95% CI** | **P – value** |  |
| Age group |  |  |  |  |  |  |  |
| - <65 years *(reference)* |  |  |  |  |  |  |  |
| - 65 – 74 years | 0.79 | 0.42 – 1.47 | 0.46 | 0.66 | 0.33 – 1.31 | 0.23 |  |
| - ≥75 years | 1.47 | 0.84 – 2.59 | 0.17 | 0.95 | 0.46 – 1.94 | 0.89 |  |
| CHA_2_DS_2_-VASc score *(per 1 unit increase)* | 1.15 | 1.005 – 1.31 | 0.043 | 0.96 | 0.80 – 1.15 | 0.67 |  |
| High bleeding risk* | 2.70 | 1.65 – 4.43 | <0.001 | 1.81 | 1.02 – 3.21 | 0.043 |  |
| Hypertension | 2.06 | 1.08 – 3.93 | 0.028 | 1.85 | 0.93 – 3.66 | 0.077 |  |
| Anemia | 2.33 | 1.52 – 3.58 | <0.001 | 1.78 | 1.12 – 2.83 | 0.014 |  |
| History of minor bleeding | 2.34 | 1.51 – 3.61 | <0.001 | 1.94 | 1.23 – 3.06 | 0.004 |  |
| History of major and/or CRNM bleeding | 3.97 | 2.28 – 6.90 | <0..01 | 2.77 | 1.53 – 5.02 | 0.001 |  |
| #Safety outcomes: Composite of major and/or CRNM bleeding.  *High bleeding risk = HAS-BLED score ≥3.  CHA2DS2-VASc score system = congestive heart failure, hypertension, age ≥75 (2 points), diabetes, stroke (2 points), vascular disease, age 65-74, sex category (female).  CI = confidence interval; CRNM = clinically relevant non-major bleeding. | | | | | | |  |

**Supplementary table 7. Univariate and multivariate analysis of the efficacy outcomes among study population^#^**

|  | **Univariable analysis** | | | **Multivariable analysis** | | | |
| --- | --- | --- | --- | --- | --- | --- | --- |
| **Variable** | **Odds ratio** | **95% CI** | **P – value** | **Odds ratio** | **95% CI** | **P – value** |  |
| CHA_2_DS_2_-VASc score *(per 1 unit increase)* | 1.25 | 1.03 – 1.51 | 0.022 | 0.95 | 0.74 – 1.20 | 0.67 |  |
| High bleeding risk* | 2.72 | 1.36 – 5.43 | 0.004 | 1.60 | 0.72 – 3.55 | 0.24 |  |
| Previous stroke and/or TIA | 3.64 | 1.93 – 6.86 | <0.001 | 3.09 | 1.41 – 6.76 | 0.005 |  |
| DOAC dosages |  |  |  |  |  |  |  |
| - Rivaroxaban 20 mg OD *(reference)* |  |  |  |  |  |  |  |
| - Rivaroxaban 15 mg OD | 1.63 | 0.52 – 5.08 | 0.39 | 1.65 | 0.51 – 5.28 | 0.39 |  |
| - Apixaban 5 BID | 1.75 | 0.68 – 4.50 | 0.24 | 1.67 | 0.64 – 4.32 | 0.29 |  |
| - Apixaban 2.5 BID | 5.39 | 2.02 – 14.36 | 0.001 | 4.64 | 1.63 – 13.20 | 0.004 |  |
| - Edoxaban 60 OD | 3.20 | 1.18 – 8.71 | 0.022 | 3.03 | 1.10 – 8.30 | 0.031 |  |
| - Edoxaban 30 OD | 5.39 | 1.35 – 21.41 | 0.017 | 4.18 | 1.01 – 17.27 | 0.048 |  |
| #Efficacy outcomes: Composite of ischemic stroke, TIA, and /or systemic embolism.  *High bleeding risk = HAS-BLED score ≥3.  CHA2DS2-VASc score system= congestive heart failure, hypertension, age ≥75 (2 points), diabetes, stroke (2 points), vascular disease, age 65-74, sex category (female).  CI = confidence interval; TIA = transient ischemic attack; DOAC = direct oral anticoagulant. | | | | | | |  |
